# Landscape, Human Disturbance, and Climate Factors Drive the Species Richness of Alien Invasive Plants on Subtropical Islands

**DOI:** 10.3390/plants13172437

**Published:** 2024-08-31

**Authors:** Yanqiu Xie, Hui Huang, Xinran Xie, Jingyao Ou, Zhen Chen, Xiaoxue Lu, Deyi Kong, Liebo Nong, Manni Lin, Zhijun Qian, Yue Mao, Ying Chen, Yingxue Wang, Zujian Chen, Chuanyuan Deng

**Affiliations:** 1 College of Landscape Architecture and Art, Fujian Agriculture and Forestry University, Fuzhou 350100, China; yanqiuxiefafu@163.com (Y.X.); zujian.chen@fafu.edu.cn (Z.C.); 2 College of Architecture and Civil Engineering, Fujian College of Water Conservancy and Electric Power, Sanming 365000, China

**Keywords:** island biogeography, life form, species–area relationship, small island effect, Fujian

## Abstract

Invasive alien plants (IAPs) pose a significant threat to island biodiversity and severely impact ecosystems. Understanding the species–area relationship and environmental determinants of growth forms for IAP species on subtropical islands is crucial for establishing an IAP’s early warning mechanism, enhancing island ecological management, and protecting the ecosystems of Fujian and other subtropical islands. The study identified significant species–area relationships for IAPs and different life-form plants (trees, shrubs, and herbs), with slopes of 0.27, 0.16, 0.15, and 0.24, respectively. The small island effect does not apply to all species. Isolation has little effect on species richness, and the IAPs on Fujian islands do not conform to the isolation effect in island biogeography. Landscape factors are the main determinants of IAPs and different life-form species richness, with area, shape index, and perimeter–area ratio being the three primary landscape factors. These environmental factors are closely related to habitat heterogeneity. Besides landscape factors, different life forms respond differently to environmental factors. Climate drives the species richness distribution of shrubs and herbs, while trees are mainly influenced by human activities. Overall, landscape, human disturbance, and climate jointly drive the distribution of IAPs, with landscape factors being the most significant.

## 1. Introduction

Invasive alien plants (IAPs) are severely threatening biodiversity, the economy, agriculture, ecosystem services, and human health, causing approximately $1.288 trillion in economic losses over the past few decades [1,2]. China, the third largest country in the world, offers suitable habitats for many alien plants from around the globe, enabling them to settle and potentially become invasive [3]. Additionally, with the increase in foreign trade and exchanges, the number and impact of IAPs are rapidly increasing, posing severe ecological threats and economic losses to China [4]. Therefore, identifying the driving factors that determine the success of plant invasions is an urgent task for controlling invasive plants.

Although islands occupy only 3–5% of the Earth’s land area, they harbor 15–20% of terrestrial species. Due to their small size and isolation [5], islands have become ideal locations for biodiversity conservation and research. However, because of their lower species richness and competitiveness [6], islands are more susceptible to invasions by alien species compared to continents [7]. The likelihood of island species being driven to extinction by humans is 12 times higher than that of continental species [8]. Therefore, to prevent the decline of native species and enhance the effectiveness of strategies for controlling and managing invasive species, it is essential to study IAPs on islands and to determine their distribution patterns.

The study of island biodiversity and the driving factors of its spatial heterogeneity originated from the traditional theory of island biogeography [9]. Previous studies have shown that a larger island area is an important factor supporting the increase in species richness of IAPs [10,11]. The island species–area relationship (ISAR) also describes the phenomenon where species richness on islands increases with the sampling area [10,11]. Besides area, factors such as isolation [12,13], island shape, index, and perimeter–area ratio related to habitat heterogeneity [14,15], altitude [16,17], temperature [18,19,20], precipitation [21], and wind speed [22] also affect the species richness of islands [19,23].

Simultaneously, frequent human activities have altered the biodiversity patterns predicted by traditional island biogeography theory [24]. Human activities (such as travel, island development, and changes in land use types) have facilitated the colonization of IAPs on islands [25] and have become significant driving factors in the changes in island species’ geographic patterns [26].

In addition to environmental factors, differences in species functional traits may also significantly impact species richness [27]. Comparative studies on the species richness of different growth forms of IAPs can reveal their responses to specific environmental factors, spatial utilization, and potential competitive relationships within communities [28]. The factors influencing the distribution of IAPs on islands are complex and varied. Therefore, examining the impact of environmental factors on the species richness of different growth forms of IAPs can offer crucial insights into how the area, landscape, climate, and human disturbance affect their distribution patterns.

The islands of Fujian belong to a subtropical region, containing a large number of endangered and endemic plants, making them a key area for the conservation and utilization of island biodiversity and species resources [29,30]. Despite the rich biodiversity of Fujian islands, diverse and complex human and socio-economic activities have led to high levels of human disturbance and multiple introductions of non-native species over centuries, causing extensive changes in the composition of flora and fauna [31]. Fujian’s numerous islands, with a wide range of areas, isolation distances, and diverse landscape features such as island shapes, coastline lengths, and elevations, make it an ideal region for studying island plant distribution and environmental driving factors [32]. Located in a subtropical maritime monsoon climate zone, Fujian reflects the plant diversity characteristics of subtropical islands in China. The suitable climate and frequent exchanges brought by economic development provide pathways for the successful invasion of alien plants, threatening the biodiversity and ecosystems of the islands [33,34]. Therefore, understanding the distribution patterns of IAPs on subtropical islands under the influence of environmental factors will provide essential information for relevant departments to predict and manage IAPs on subtropical islands.

Currently, the distribution patterns of IAPs and the species richness of different growth forms on Fujian islands are not well understood, and environmental factors at different scales, as well as different growth forms, may influence the results. We address this issue by answering the following questions: (1) Do the species richness distributions of IAPs and different growth forms conform to island biogeography theory? (2) Besides being influenced by landscape environmental factors such as area and isolation, are IAPs and different growth forms also affected by climate and human disturbance? (3) Among the three types of environmental factors, which is the most critical driving factor for the spatial distribution of IAPs? To address these questions, this study focuses on 53 islands in Fujian. First, we describe the composition of IAPs. Second, we discuss the species–area relationships of IAPs and different growth forms. Finally, we analyze the role of ten environmental factors representing landscape, climate, and human disturbance in driving the species richness of IAPs and different growth forms to explain the distribution patterns of IAPs and different growth forms on Fujian islands.

## 2. Materials and Methods

### 2.1. Study Area

**Figure 1:**
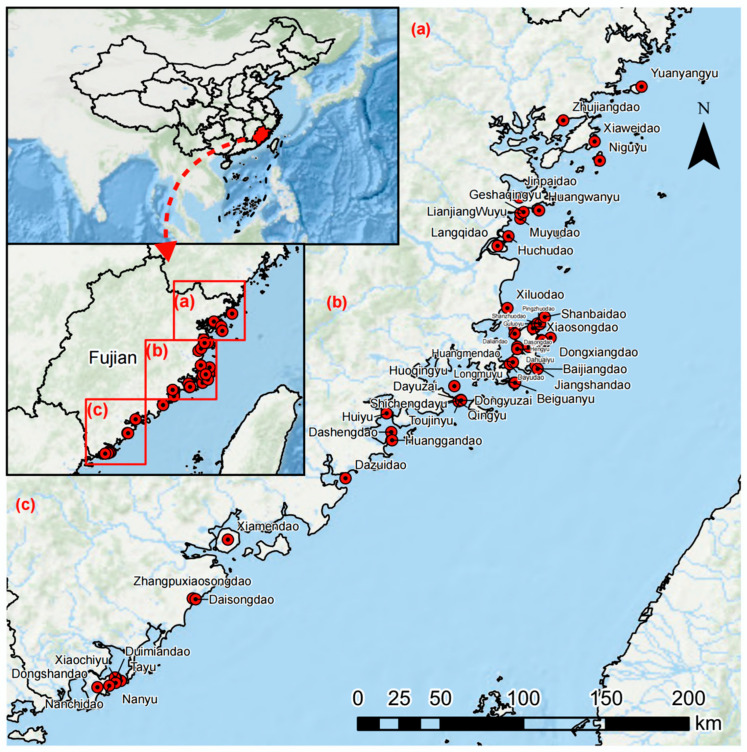
Location map of the 53 islands in Fujian (Islands 1–53 are listed in Table A1).

Fujian Province is located in the southeastern coastal region of China, near the Taiwan Strait. The islands of Fujian are all continental islands, with terrain primarily consisting of hills, terraces, plains, and aeolian sand areas. The main soil types are lateritic red soil, red soil, sandy soil, and coastal saline soil. The climate is a subtropical maritime monsoon climate; south of the Min River estuary is a southern subtropical maritime monsoon climate, and north of the Min River estuary is a mid-subtropical maritime monsoon climate [35]. The annual average temperature ranges from 15.2 to 21.0 °C, and the annual average precipitation ranges from 1000 to 1600 mm. The main types of island vegetation are evergreen coniferous forests, evergreen broad-leaved forests, and shrub grass communities. There are over 1500 islands in Fujian, but many are small, vegetated outcropping reefs. The islands are scattered and mostly uninhabited, making access difficult. Therefore, this study selected islands within the Fujian Sea area (117°31′61″–120°68′66″ E, 23°60′90″–26°93′08″ N) that are accessible and vegetated. To reflect differences in landscape, climate, and human disturbance, the study selected islands with environmental gradients, including variations in area, elevation, degree of isolation, human activity levels, and climatic conditions. We selected a total of 53 islands (Figure 1), including 44 uninhabited islands such as Tayu, Duimianyu, Huanggan Island, Dazui Island, and Huangwan Island, and 9 inhabited islands such as Haitan Island, Huiyu, Langqi Island, Dalian Island, and Daisong Island. The average area of the islands is 13.39 km^2^, with the smallest being only 0.007 km^2^ and the largest being 267.13 km^2^ (Haitan Island). Of these, 84.90% (45 islands) have an area of less than 1 km^2^. The average elevation of the islands is 70.34 m, ranging from 13.98 to 438.20 m.

### 2.2. Plant Species Data

Field surveys were conducted on 53 islands in Fujian from 2018 to 2022. To comprehensively record the plant species on each island, surveys were conducted twice during the key growing seasons in the Fujian region (May–July and September–November). During these periods, plant species and numbers peak, maximizing the accuracy and completeness of species records. The primary survey method was transect surveys, supplemented by quadrat surveys. Additionally, drone aerial photography was used to assist in inaccessible areas such as island edges and cliffs. For small islands with an area not exceeding 1 km^2^ (n = 45), a complete island survey was conducted: 3–10 transects were laid out on each island, and each transect was walked at least twice [36]. On islands with an area of 1–10 km^2^ (n = 3), 6–20 transects were established on each island, with a total transect length of more than 12 km per island. On islands larger than 10 km^2^ (n = 5), the same survey method was applied, with additional transects centered on prominent mountains on each island [37], with a total transect length of more than 25 km per large island. Detailed species information within the transects was recorded.

To ensure comprehensive species records, after the transect surveys, the typical community types of each island were identified, and standard plot methods were used to set up quadrats covering different types of plant communities [38,39,40]. Based on the typical plant communities of the islands, 20 m × 20 m forest plots were established, with a total of 675 quadrats on 53 islands. Detailed information for each quadrat, including latitude and longitude, elevation, slope, terrain, canopy density, and habitat type, was recorded [41]. Photos were taken on-site, and specimens were collected. Voucher specimens are stored in the herbarium of the College of Landscape Architecture and Art at Fujian Agriculture and Forestry University.

All species were identified according to the Flora of China (FOC http://www.efloras.org (accessed on 5 January 2022)). Invasive alien plant species were screened and identified using the China Invasive Alien Species Information System (https://www.iplant.cn/ias/ (accessed on 21 September 2022)). Additionally, plants were classified into three growth forms: trees, shrubs, and herbs. Vines were not classified separately; woody vines were included with shrubs, and herbaceous vines were included with herbs [42].

### 2.3. Environmental Factors

To analyze the impact of island environmental factors on the species richness of IAPs and different growth forms, 10 variables were selected from three categories: landscape, climate, and human disturbance.

Area, elevation, distance to the mainland (DM), distance to the nearest big island (DN), perimeter–area ratio (PAR), and shape index (SI) are the six variables selected as landscape factors. Area is the most important parameter of an island, determining its space, population carrying capacity, and ecological processes [9]. Distance to the mainland (DM) and distance to the nearest big island (DN) are common factors influencing the frequency of visits by residents and tourists, and they are quantitative indicators of island isolation [43]. The island’s area and perimeter were used to generate the perimeter–area ratio and shape index. The perimeter–area ratio is the ratio of the island’s edge to its area, indicating the relative amount of edge habitat compared to interior habitat, representing the edge effect of the island [44]. The shape index represents the complexity of the island’s shape; when the island’s shape is circular, its shape index is 1. As the shape becomes more complex and irregular, the shape index increases. The formula for SI is SI = P/[2 × (π × A)^0.5^] [45], where P is the island’s perimeter, and A is the island’s area.

Annual mean wind (AMW), annual mean temperature (AMT), and annual precipitation (AP) are the three variables selected as climate factors. Annual mean wind data were sourced from the NOAA 10 m resolution wind vector map (https://www.noaa.gov (accessed on 7 October 2022)), and annual mean temperature and annual precipitation data were sourced from the global bioclimatic variables of WorldClim (http://www.worldclim.org (accessed on 23 November 2022)). Based on the latitude and longitude of the Fujian islands, the climate data for the 53 islands were extracted using Kriging interpolation.

The proportion of buildings and farmland area (BFA) is the factor selected for human disturbance. BFA is the ratio of the combined area of buildings and farmland to the total area of the island. Distance to the mainland (DM) and distance to the nearest big island (DN) are used to represent the degree of isolation of the islands [36].

The area and perimeter of the islands, distance to the mainland, distance to the nearest big island, and proportion of buildings and farmland area data were sourced from the 2020 global 30 m resolution land cover vector map published by the Aerospace Information Research Institute, Chinese Academy of Sciences (https://data.casearth.cn (accessed on 2 December 2022)). The “mask extraction” method was used to extract the area, perimeter, and land use type area for each island, and to calculate the building and farmland coverage area. Elevation data were sourced from the 30 m resolution digital elevation model map from the Geospatial Data Cloud (http://www.gscloud.cn (accessed on 3 June 2022)). The “mask extraction” tool was used to obtain the raster range of each island and extract the elevation of the highest point on each island. All the above tasks were completed using ArcGIS 10.8.

### 2.4. Statistics and Analysis

Before constructing the model, the Variance Inflation Factor (VIF) was used to detect collinearity among the environmental factors. The VIF for elevation among the 10 environmental factors was greater than 5, so elevation (EL) was excluded as an environmental factor. The VIF for the remaining 9 environmental factors was less than 5, indicating weak collinearity [46]. Species accumulation curves were used to determine whether the survey of IAPs on Fujian islands was sufficient.

Using the sars package in R (Version 4.1.1), a log-transformed power function model logS = logc + z × logA was employed to represent the species–area relationship between the species richness of IAPs, different growth forms, and island area. In this model, S represents species richness, A represents island area, and c and z are constants. The parameter z indicates the rate at which species richness changes with area and is commonly used to measure the sensitivity of species and communities to fragmented habitats [36].

Pearson correlation coefficients were used to detect correlations among island environmental factors, with significance levels adjusted using the Bonferroni correction. Global Moran’s I was employed to detect spatial autocorrelation of environmental factors, and Mantel tests were used to analyze the correlation between the environmental factor matrix and the presence-absence matrix of IAPs and different growth forms based on Euclidean distance.

Global Moran’s I analysis indicated that the distance to the mainland (DM) (Moran’s I = 0.90, *p* < 0.001) has highly significant spatial autocorrelation. The Mantel test showed that the distance to the mainland (DM) did not have a significant effect on the species richness of IAPs and different growth forms (*p* > 0.05). Therefore, the distance to the mainland (DM) was retained as an environmental factor.

To explore the impact of island environmental factors on the species richness of IAPs and different growth forms, both the independent and dependent variables were standardized. Full subset regression was employed, with subsets ranked according to the corrected Akaike Information Criterion for small samples (AICc) and other criteria. The optimal subset method was used as the standard for selecting the best model. Based on this, hierarchical partitioning was used to evaluate the environmental factors in the best-fitting model, providing a quantitative analysis of the explanatory power of different environmental variables. All the above data analyses were completed using R version 4.2.2.

## 3. Results

### 3.1. Analysis of Species Composition and Distribution Patterns

Among the 53 islands in Fujian, there are 130 species of IAPs, spanning 99 genera and 38 families (including subspecies, Appendix A Table A2). This includes 12 tree species, accounting for 9.23% of the total; 17 shrub species, accounting for 13.08%; and 101 herb species, accounting for 77.69%. At the island scale, Haitandao has the highest species richness (63), while Toujinyu and Dongzhuodao have a species richness of 0. This does not mean that there are no plants on these islands, but rather that their small size lacks IAPs. However, they still hold statistical significance and were included in the analysis.

**Figure 2:**
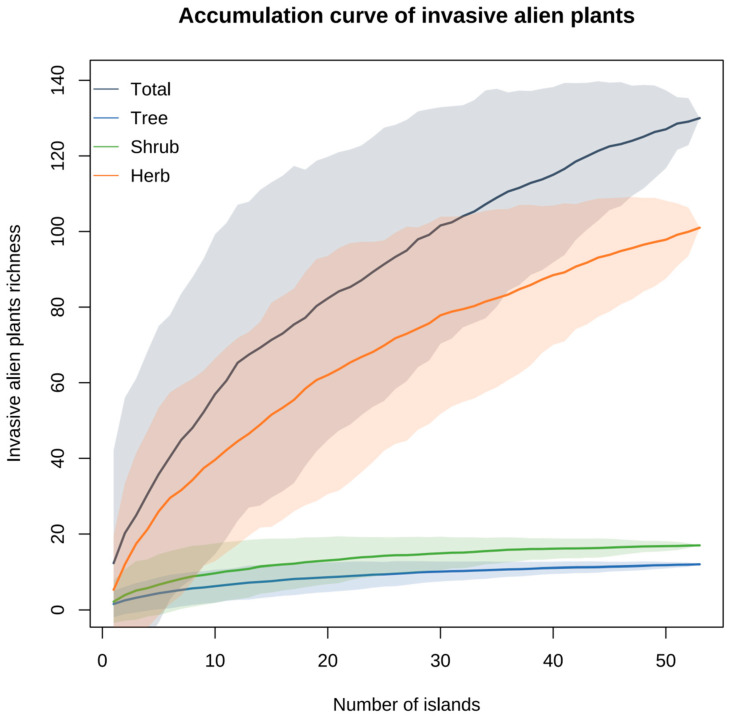
Species accumulation curves of IAPs and different growth forms on Fujian islands.

The species accumulation curve (Figure 2) indicates that as the number of islands increases, both the total number of invasive alien plant species and the number of herb species initially rise rapidly, followed by a slower rate of increase. This suggests that in the early stages, the number of invasive alien plant species discovered increases rapidly with the number of islands surveyed, but once the number of islands reaches a certain threshold, the rate of new species discovery slows down. Although the curves for tree and shrub species are relatively flat due to their lower numbers on the islands, the overall data are reasonable, indicating that the survey of IAPs on the 53 islands was sufficient.

### 3.2. Species–Area Relationship

**Figure 3:**
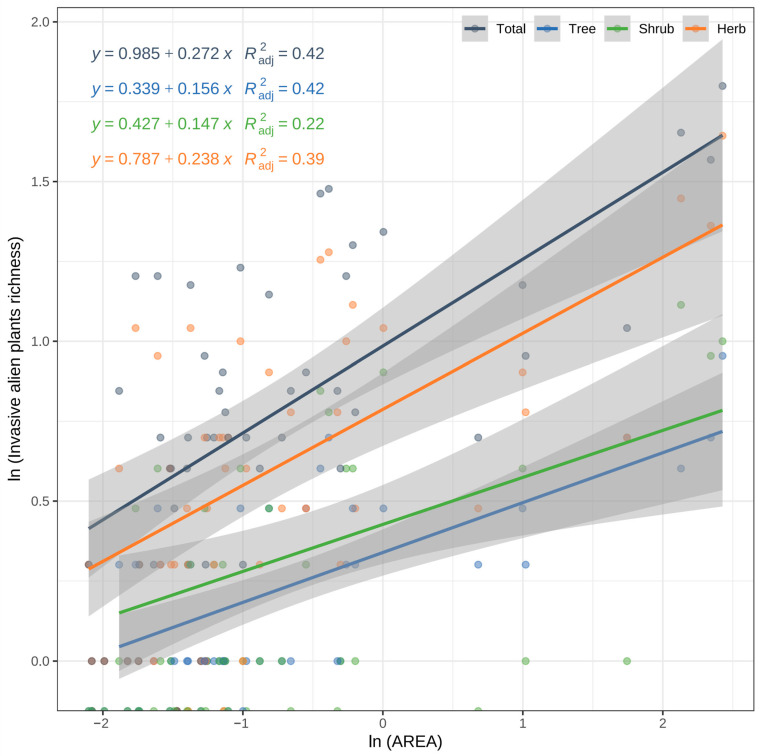
Species–area relationships of IAPs and different growth forms.

The species–area relationships for IAPs, trees, shrubs, and herbs all show a positive correlation, indicating that the species richness of IAPs increases with the area (Figure 3). The explanatory power of area for species richness is as follows: total IAPs 42%, tree 42%, shrub 22%, and herb 39%.

A larger slope indicates a faster increase in species richness with the island area. The slope (z-value) for the total IAPs is the largest (0.27), followed by herb (0.24), with tree (0.16) and shrub (0.15) being smaller. Overall, the larger the island area, the higher the species richness of IAPs, with this relationship being most significant for total IAPs. The relationship between species richness and island area for trees and shrubs is relatively weaker but still shows a positive correlation.

### 3.3. IAPs and Island Environmental Factors

**Figure 4:**
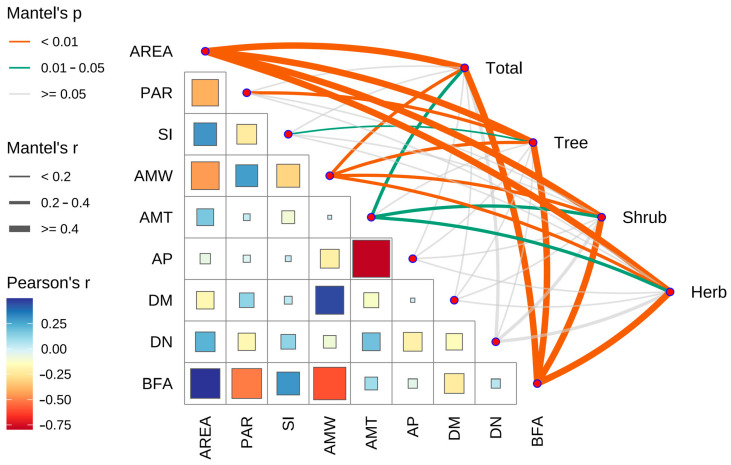
Pearson correlation analysis and Mantel test between environmental factors. Area: area; PAR: perimeter–area ratio; SI: shape index; AMW: annual mean wind speed; AMT: annual mean temperature; AP: annual precipitation; DM: distance to the mainland; DN: distance to the nearest big island; BFA: proportion of buildings and farmland area. The colors in the matrix represent the size and direction of the Pearson correlation coefficient (r-value). Red indicates a positive correlation, and blue indicates a negative correlation. The deeper the color, the stronger the correlation. The size of the squares indicates the significance of the correlation. Larger squares represent more significant correlations (smaller *p*-values). In the network diagram, the color of the lines represents the *p*-value of the Mantel test; orange: *p* < 0.01; green: 0.01 ≤ *p* < 0.05; gray: *p* ≥ 0.05. The thickness of the lines represents Mantel’s r-value (correlation coefficient), with thicker lines indicating stronger correlations.

According to the correlation analysis of island environmental factors (Figure 4), area is an important environmental factor that shows significant correlations with several other factors. Specifically, area is significantly positively correlated with the proportion of buildings and farmland area (BFA) (r = 0.50, *p* < 0.001) and shape index (SI) (r = 0.30, *p* < 0.05), and significantly negatively correlated with perimeter–area ratio (PAR) (r = −0.40, *p* < 0.01) and annual mean wind speed (AMW) (r = −0.45, *p* < 0.001).

The significant negative correlation between perimeter–area ratio (PAR) and the proportion of buildings and farmland area (BFA) (r = −0.51, *p* < 0.001) further indicates a close relationship between the shape of the island and its level of development. A higher perimeter–area ratio typically corresponds to a more irregular island shape, and these islands may be less developed.

The significant negative correlations between annual mean wind speed (AMW) and both area and BFA (*p* < 0.001) indicate that islands with larger areas and higher BFA typically have lower wind speeds. Additionally, the significant positive correlation between annual mean wind speed and distance to the mainland (DM) suggests that islands farther from the mainland may experience stronger winds.

According to the Mantel analysis of the correlation between island environmental factors and the species richness of IAPs, area is identified as a key environmental factor, significantly positively correlated with the species richness of IAPs. Additionally, the climate factors of annual mean temperature (AMT) and annual precipitation (AP) significantly impact the species richness of IAPs. The species richness of trees and herbs shows a more pronounced response to these environmental factors, while the species richness of shrubs shows a weaker response.

**Figure 5:**
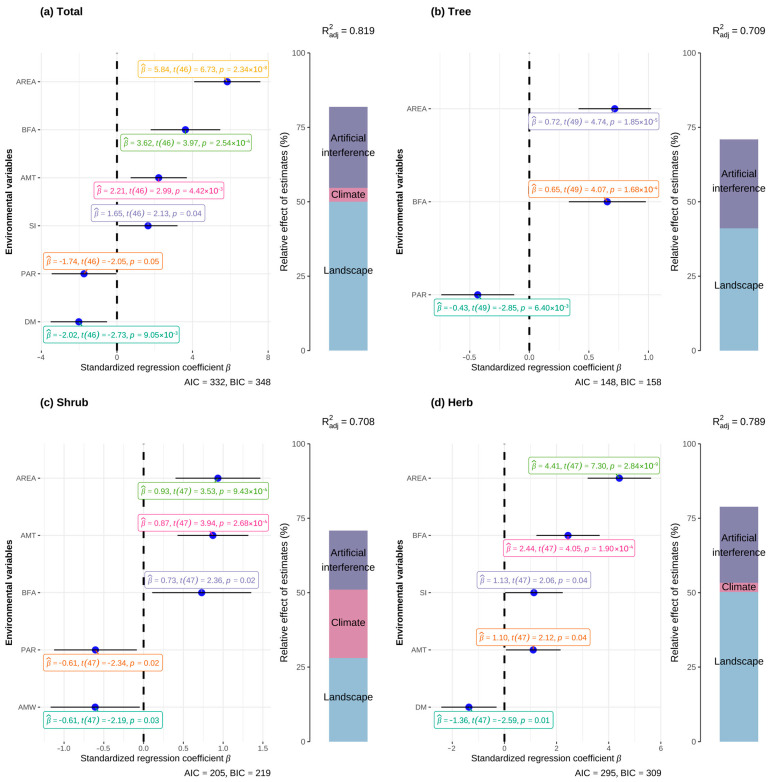
Optimal model selection and hierarchical partitioning analysis for total species richness. The left side of the figure presents the optimal model obtained using full subset regression, selected based on the minimum AIC value. Negative correlations are depicted to the left of the dashed line, and positive correlations to the right. The right side of the figure illustrates the hierarchical partitioning analysis based on the optimal model.

The results of hierarchical partitioning (Figure 5) show that the selected environmental factors effectively explain the distribution patterns of species richness for IAPs and different growth forms. The environmental factors have the highest explanatory power for the species richness of IAPs, reaching 81.9%.

Landscape factors played a major role in explaining species richness, while the relative contributions of human disturbance and climate factors varied according to plant growth forms. Specifically, landscape factors provided more explanatory power compared to climate and human disturbance factors (total species richness 61.10%, trees 57.93%, shrubs 39.63%, herbs 63.64%). Human disturbance factors followed, explaining 33.20%, 42.12%, 27.97%, and 32.38% of the species richness for total IAPs, trees, shrubs, and herbs, respectively. Climate factors contributed to the total species richness of IAPs (5.69%), shrubs (32.46%), and herbs (3.93%), but did not significantly impact tree species richness.

Simultaneously, based on the optimal model selected by full subset regression (Figure 5), it can be observed that area, shape index (SI), and perimeter–area ratio (PAR) among the landscape factors are the primary factors affecting the total species richness and the species richness of different growth forms.

Landscape factors such as area (area), perimeter–area ratio (PAR), and shape index (SI) played a major role in explaining species richness. Area had a significant positive impact on the total species richness and the species richness of different growth forms. In contrast, the species richness of IAPs, trees, and shrubs decreased with an increase in the perimeter–area ratio (PAR), while herb species richness was not affected by the perimeter–area ratio (PAR). Shape index (SI) influenced the total species richness and herb species richness, with both increasing as the shape index (SI) increased. Isolation also affected the total species richness and herb species richness, with both decreasing as the distance to the mainland (DM) increased. Human disturbance factors, such as the proportion of buildings and farmland area (BFA), had a significant positive impact on the species richness of all types of plants, with species richness increasing significantly as BFA increased. The impact of climate factors such as annual mean temperature (AMT) and annual mean wind speed (AMW) on species richness varied by plant type: shrub species richness decreased as AMW increased. AMT had a significant positive impact on the total species richness, as well as the species richness of shrubs and herbs.

## 4. Discussion

The small-island effect (SIE) is an important theory in biogeography that describes the relationship between island area and species diversity. According to MacArthur and Wilson’s [9,47] island biogeography theory, small islands typically have lower species richness due to their small area, limited habitat types, scarce resources, and higher extinction rates. In contrast, larger islands support more species because of their larger area, diverse habitat types, and abundant resources. This theory has been validated in multiple island ecosystems [15,48,49,50].

This study found that the species richness of IAPs on 53 Fujian islands increases with island area, particularly for herbaceous plants, which showed the most significant response to area changes (z-value of 0.24), consistent with the predictions of the small-island effect [51,52]. However, the study also found that the species–area relationship slopes (z-values) for trees and shrubs were lower, at 0.16 and 0.15, respectively, below the typical z-value range (0.20–0.35) [37,52]. This suggests that the distribution of trees and shrubs on Fujian islands does not fully conform to the small-island effect theory. In this study, the low z-values for trees and shrubs may be due to the weaker dispersal and settlement abilities of the primary tree species (*Casuarina equisetifolia* L., *Acacia confusa* Merr.) and shrub species (*Malvastrum coromandelianum* (L.) Garcke and *Sida szechuensis* Matsuda) compared to the dominant herbaceous IAPs (such as *Bidens pilosa* L., *Symphyotrichum subulatum* (Michx.) G.L.Nesom, *Erigeron canadensis* L. of Asteraceae, and *Alternanthera philoxeroides* (Mart.) Griseb. of Amaranthaceae) on the 53 Fujian islands.

This study found that the species richness of IAPs on the 53 islands in Fujian increases with island area, particularly for herbaceous plants, which show the most significant response to changes in area (z-value of 0.24). This is consistent with the predictions of the small-island effect [51,52]. Larger islands provide more habitats and niches, supporting more species, which is the primary reason for the increased species richness of herbaceous plants. However, the study also found that the species–area relationship slopes (z-values) for trees and shrubs were lower, at 0.16 and 0.15, respectively, below the typical z-value range (0.20–0.35) [37,52]. This suggests that the distribution of trees and shrubs on Fujian islands does not fully conform to the small-island effect theory. The small-island effect is generally more pronounced on smaller islands, but in this study, the low z-values for trees and shrubs may be due to their lower adaptability to environmental changes and weaker dispersal and settlement abilities.

The applicability of the small-island effect varies among different types of plants. Herbaceous plants, due to their strong dispersal and adaptation abilities, are more likely to establish on larger islands, making their species–area relationship more pronounced. In contrast, trees and shrubs, with longer growth cycles, higher environmental requirements, and weaker dispersal abilities, exhibit a less significant species–area relationship. This also confirms the findings of Lomolino [53] and Sanderson et al. [54], which state that the small-island effect does not apply to all species, particularly those with differing environmental adaptability and dispersal abilities.

In island biogeography research, isolation is widely regarded as a major factor influencing species richness [55,56]. However, this study found that the species richness of IAPs on Fujian islands was not as closely related to isolation as predicted by theory. After analyzing the environmental factors driving the species richness of IAPs and different growth forms, it was found that isolation had a low importance for the species richness of IAPs. This may be due to the relatively short distances between the islands and the nearest mainland or large island (the maximum distances being 26.50 km and 40.97 km, respectively). Such small degrees of isolation are insufficient to limit the spread of most invasive alien species. Isolation is not the main barrier to the distribution of IAPs. With increasing human activity, DM and DN are not suitable indicators of island isolation. Studies based on the habitat islands of Qiandao Lake and the Shengsi Archipelago also found that isolation does not affect species richness [37,42]. These studies suggest that distance is not strongly correlated with the species richness of IAPs and different growth forms on islands. Instead, the species richness of IAPs and different growth forms on islands may be more influenced by landscape, human activities, and climatic conditions.

In discussing the driving factors of species richness of IAPs in Fujian, hierarchical partitioning analysis identified the combined effects of landscape, human disturbance, and climate factors. The results showed that although all three factors influence the species richness of IAPs and different growth forms, landscape factors contribute the most, with area playing a dominant role in determining species richness. This finding is consistent with the hypothesis in island biogeography theory, which states that area is the main factor determining species richness [18]. The foundation of island biogeography theory is the species–area relationship proposed by MacArthur and Wilson [47], which indicates that larger islands can support more species. This is because larger islands can provide more habitat types and niches, as well as accommodate larger populations, reducing the probability of species extinction [10]. The results of this study further validate this theory, particularly in the context of IAPs, where the decisive role of area in determining species richness is even more significant.

Simultaneously, the shape index, representing the complexity of island shapes, and the perimeter–area ratio, representing the relative amount of edge habitat compared to interior habitat, are factors closely related to IAPs. In the correlation analysis of island environmental factors (Figure 4), the positive correlation between island area and shape index indicates that larger islands have more complex shapes. This means that larger islands not only have expanded areas but also more edge habitats, providing more living space for species with different niches. Additionally, the negative correlation between island area and perimeter–area ratio suggests that as island area increases, the complexity of the island shape increases, resulting in more relative interior habitat. This finding indicates that larger islands, due to their more complex shapes (higher shape index) and more interior habitats (lower perimeter–area ratio), represent higher habitat heterogeneity, supporting higher species richness. This also indirectly reflects the positive correlation between area, shape index, and perimeter–area ratio with habitat heterogeneity [36]. This conclusion aligns with previous research, indicating that species diversity increases with area as habitat heterogeneity increases [53]. Furthermore, a study of 700 islands worldwide found that environmental factors representing habitat heterogeneity are the determining factors affecting species richness [28].

Among human disturbance factors, the proportion of buildings and farmland area (BFA) has a significant positive impact on the species richness of IAPs and different growth forms. Their species richness increases significantly with the increase of BFA. This may be because human activities increase opportunities for species introduction and habitat diversity. Human activities, such as agriculture and urban construction, often lead to land use changes that create new habitats and increase the introduction and spread of plant species. For example, agricultural activities can introduce alien species through sowing and transplanting, while also promoting species colonization and spread by altering soil and hydrological conditions [57]. Additionally, urban construction can introduce various plants through horticulture, landscaping, and transportation. These activities increase the mixing of native and alien plant species, thereby enhancing the species richness of IAPs [58,59].

Different growth forms of IAPs respond differently to climate factors. Specifically, in this study, annual mean temperature (AMT) had a significant positive impact on the species richness of IAPs, as well as shrubs and herbs. This may be because warmer climates can extend the growing season, increase growth rates, and improve reproductive success [60,61]. Studies have shown that warm climates can enhance photosynthesis and nutrient uptake in plants, thereby increasing their growth and dispersal abilities [62]. This study found that the species richness of shrubs decreased with an increase in annual mean wind speed (AMW), possibly due to the mechanical damage caused by high wind speeds, which affects water use efficiency and growth [63]. Additionally, strong winds may limit shrub dispersal by affecting seed distribution and germination [64]. Herbaceous plants did not show a significant response to wind speed, likely due to their high adaptability, allowing them to quickly complete their life cycle and maintain high reproductive success rates [65]. Furthermore, the species richness of trees was not influenced by climate factors, whereas BFA had a significant positive effect on tree species richness, indicating that human activities play an important role in the introduction and spread of trees. This is because trees typically require longer growth periods and stable environments, and human activities can provide continuous habitat support and resource supply, promoting tree colonization and growth.

Overall, compared to landscape factors, human disturbance factors, and climate factors, the explanatory power for species richness of IAPs and different growth forms is lower. This may be partly related to the climate factors (annual mean wind speed, annual mean temperature, annual precipitation) and human disturbance factors (proportion of buildings and farmland area) used in this study, which only describe certain aspects of the climate and disturbances affecting the distribution of endemic plants, possibly neglecting the seasonal or interannual variability of environmental factors [66]. Additionally, for islands with complex terrain and rich habitat heterogeneity, these environmental factors may change drastically within small areas, highlighting a limitation of the environmental data used in this study. Therefore, future research could incorporate more specific and detailed factors such as climate, human disturbance, and soil physical and chemical properties, which may help to more comprehensively and deeply understand the distribution patterns of invasive alien plant species richness on Fujian islands and other subtropical islands.

## 5. Conclusions

This research demonstrates how different growth forms of IAPs on Fujian islands respond to landscape, climate, and human disturbance factors, and identifies key factors influencing island plant species richness. Our findings indicate that the species richness of IAPs on islands is driven by various environmental factors. Additionally, the factors influencing species richness vary across different growth forms. We observed a significant species–area relationship for IAPs and different growth forms on Fujian islands, consistent with island biogeography theory. The low z-values for trees and shrubs confirm that the small-island effect does not apply to all species. Furthermore, unlike traditional island biogeography studies, isolation was not the primary barrier to the distribution of IAPs. Increased tourism, road and bridge construction, and frequent human activities suggest that DM and DN might not be the most suitable indicators of island isolation. However, there are currently no suitable indicators to assess these impacts. Further research is needed to understand changes in island isolation caused by human activities. Notably, landscape, human disturbance, and climate factors jointly drive the species richness of IAPs on Fujian islands, with landscape being the primary factor. Specifically, area, shape index, and perimeter–area ratio are the three most important determinants of species richness for IAPs and different growth forms on Fujian islands, closely related to habitat heterogeneity. Meanwhile, different growth forms of IAPs (trees, shrubs, and herbs) respond differently to environmental factors due to variations in plant traits and adaptability to environmental pressures and human disturbances. Climate primarily drives the species richness distribution of shrubs and herbs but has a weaker effect on trees. The proportion of buildings and farmland area (BFA) has a significant positive effect on tree species richness, indicating that tree species richness is mainly influenced by human activities, whereas climate plays a role in driving the distribution of shrubs and herbs. Understanding the impact of island environmental factors on the species richness of IAPs and different growth forms is crucial for subsequent efforts in the conservation and sustainable use of invasive alien plant resources. Future research should incorporate more specific climate, human disturbance, and soil physicochemical properties, as well as more case studies (islands and archipelagos) and data for analysis to reveal the detailed impacts of island environmental factors on species richness and plant diversity.

## Data Availability

The data in this paper need to remain confidential for the time being and therefore can not be made public for the time being.

## Appendix A

**Table A1:** An overview of 53 islands in Fujian selected for this study.

Islands Name	Latitude and Longitude	Species Richness	No. of Forest Investigation Plots	No. of Shrub Investigation Plots	No. of Herb Investigation Plots	Area (km^2)^	Elevation (m)	Perimeter–area Ratio	Shape Index	Annual Mean Wind (m/s)	Annual Mean Temperature (°C)	Annual Precipitation (mm)	Distance to the Mainland (km)	Distance to the Nearest Large Island (km)	Proportion of Buildings and Farmland Area (%)
Baijiangdao	119°815′ E–25°425′ N	2	4	16	20	0.05	33.6	27.5	1.81	8.86	19.94	1258	17	3.92	-
Beiguanyu	119°687′ E–25°349′ N	4	6	24	30	0.13	40.90	12.94	1.33	8.22	19.94	1258	3.29	8.42	-
Beixiangluyu	119°690′ E–25°616′ N	5	3	12	15	0.04	15.6	20.83	1.18	8.44	19.84	1257	9.79	0.6	-
Chixietedao	119°789′ E–25°646′ N	2	2	8	10	0.02	28.9	31.89	1.37	7.88	19.84	1257	19.77	1.14	-
Dahuaiyu	119°705′ E–25°545′ N	3	3	12	15	0.03	26.80	31.78	1.61	6.79	19.84	1257	9.17	0.63	-
Daisongdao	117°957′ E–24°174′ N	29	12	48	60	0.36	27.30	7.71	1.3	4.81	20.96	1142	0.4	1.4	0.79
Daliandao	119°689′ E–25°649′ N	16	30	120	150	9.96	238.5	2.03	1.81	5.96	19.84	1257	7.88	1.18	0.17
Dashengdao	119°020′E–25°081′ N	1	5	20	25	0.07	442	17.78	136	7.9	20.32	1131	2.54	1.28	-
Dasongdao	119°799′ E–25°649′ N	9	10	40	50	0.28	51.80	8.86	1.33	9.06	19.84	1257	20.14	1.16	-
Dayudao	119°663′ E–25°451′ N	7	8	32	40	0.22	45.10	12.38	1.64	7.76	19.94	1211	2.47	1.05	0.13
Dayuzai	119°398′ E–25°254′ N	1	2	8	10	0.01	13.98	17.67	0.45	8.30	20.30	1190	3.33	4.38	-
Dazuidao	118°771′ E–24°830′ N	20	16	64	80	0.61	103.10	8.39	1.85	7.19	20.57	1085	2.33	40.97	0.04
Dongshandao	117°424′ E–23°698′ N	37	80	320	400	220.18	274.30	0.51	2.12	3.07	21.28	1296	0.31	23.13	0.24
Dongxiangdao	119°885′ E–25°594′ N	5	24	96	120	4.8	134.60	4.1	2.53	5.56	19.75	1280	26.5	2.3	0.46
Dongyuzai	119°400′ E–25°254′ N	1	2	8	10	0.02	16.00	29.8	1.03	8.28	20.3	1190	3.31	4.11	-
Dongzhuodao	119°828′ E–25°669′ N	0	3	12	15	0.03	36.00	27.73	1.44	7.95	20.03	1259	21.27	4.11	-
Duimiandao	117°524′ E–23°748′ N	17	6	24	30	0.10	25.80	20.71	1.81	6.3	21.22	1239	5.78	0.49	0.01
Geshaqingyu	119°738′ E–26°278′ N	2	2	8	10	0.02	21.05	42.14	1.60	4.53	18.81	1436	0.66	1.49	-
Guangyouyu	119°834′ E–25°581′ N	5	4	16	20	0.06	24.20	17.30	1.22	8.14	19.75	1280	22.57	0.32	0.03
Guluoyu	119°813′ E–25°671′ N	1	2	8	10	0.01	33.2	41.84	1.19	7.88	20.03	1259	20.33	2.64	-
Haitandao	119°766′ E–25°544′ N	63	104	416	520	267.13	438.2	0.48	2.23	4.67	19.84	1257	3.59	1.19	0.62
Hengyu	119°704′ E–25°533′ N	4	3	12	15	0.03	22.4	31.23	1.53	7.06	19.84	1257	9.04	0.47	-
Hongshanyu	119°841′ E–25°578′ N	4	3	12	15	0.03	20.8	33.09	1.63	7.98	19.75	1280	23.05	0.67	-
Huanggandao	119°024′ E–25°037′ N	16	16	64	80	0.55	72.4	8.27	1.73	6.34	20.32	1131	0.72	3.99	-
Huangmendao	119°680′ E–25°461′ N	8	4	16	20	0.07	34.7	19.73	1.49	6.85	19.94	1258	3.89	0.15	-
Huangwanyu	119°823′ E–26°287′ N	7	5	20	25	0.07	26.9	28.88	2.12	6.46	18.81	1436	1.2	1.15	0.01
Huchudao	119°656′ E–26°146′ N	6	5	20	25	0.07	33.72	22.56	1.74	5.5	19.35	1352	5.2	0.38	-
Huiyu	118°995′ E–25°183′ N	30	14	56	70	0.41	59.9	13.14	2.38	6.58	20.04	1196	1.11	1.28	0.55
Huoqingyu	119°363′ E–25°331′ N	4	16	64	80	0.5	54	9.32	1.86	8.67	20.3	1190	5.79	1.82	-
Jiangshandao	119°804′ E–25°443′ N	7	14	56	70	0.47	68.1	10.01	1.94	10.71	19.94	1258	17.33	1.12	-
Jinpaidao	119°716′ E–26°357′ N	9	4	16	20	0.05	18.82	17.27	1.12	4.53	18.54	1493	1.36	0.44	-
Langqidao	119°597′ E–26°094′ N	11	60	240	300	55.5	275	0.59	1.23	3.09	19.35	1352	0.56	2.3	0.60
LianjiangWuyu	119°721′ E–26°270′ N	5	6	24	30	0.11	41.68	16.45	1.51	5.36	18.81	1436	0.62	2.59	-
Longmuyu	119°692′ E–25°349′ N	5	3	12	15	0.03	20.2	37.97	1.72	7.59	19.94	1258	3.95	0.57	-
Muyudao	119°721′ E–26°242′ N	5	8	32	40	0.19	17.81	13.53	1.66	5.85	18.81	1436	3.64	2.52	0.01
Nanchidao	117°488′ E–23°706′ N	16	2	8	10	0.02	49.52	28.19	1.25	5.73	21.28	1296	0.05	0.03	0.01
Nanyu	117°523′ E–23°721′ N	15	3	12	15	0.04	22.7	20.09	1.17	6.39	21.22	1239	0.12	0.12	0.01
Niguyu	120°152′ E–26°558′ N	9	32	128	160	10.48	231.83	0.57	0.52	11.67	18.14	1495	10.99	0.59	-
Pingzhuodao	119°821′ E–25°668′ N	1	4	16	20	0.05	40	18.12	1.14	7.80	20.03	1259	21.09	3.38	-
Qingyu	119°385′ E–25°249′ N	2	2	8	10	0.01	20	48.63	1.22	8.20	20.30	1190	1.95	5.32	-
Shanbaidao	119°853′ E–25°707′ N	1	2	8	10	0.02	34.6	32.2	1.22	7.86	20.03	1259	22.75	8.21	-
Shanzhuodao	119°819′ E–25°673′ N	4	3	12	15	0.04	52.2	21.98	1.24	7.74	20.03	1259	20.74	3.30	-
Shichengdayu	119°398′ E–25°256′ N	2	3	12	15	0.04	31	19.09	1.09	9.01	20.3	1190	3.12	4.31	-
Tayu	117°551′ E–23°732′ N	22	20	80	100	1.01	91.3	6.82	1.93	5.53	21.22	1239	2.44	1.16	0.26
Toujinyu	119°391′ E–25°251′ N	0	2	8	10	0.01	21.1	52.37	1.35	8.22	20.3	1190	2.40	4.85	-
Xiamendao	118°132′ E–24°497′ N	45	72	288	360	134.84	339.6	0.49	1.61	2.65	20.96	1093	0.71	5.42	0.80
Xiaochiyu	117°519′ E–23°749′ N	7	2	8	10	0.01	19.2	16.85	0.54	5.83	21.22	1239	0.43	0.36	-
Xiaosongdao	119°792′ E–25°654′ N	5	4	16	20	0.06	36.8	24.17	1.61	7.87	19.84	1257	19.43	0.28	-
Xiaweidao	120°124′ E–26°661′ N	5	5	20	25	0.08	35.51	22.42	1.77	6.49	18.14	1495	0.27	2.63	-
Xiluodao	119°650′ E–25°755′ N	2	6	24	30	0.10	39.85	15.96	1.42	7.46	20.03	1259	1.74	14.24	-
Yuanyangyu	120°379′ E–26°960′ N	6	16	64	80	0.64	149.81	9.01	2.02	5.03	17.43	1406	12.85	0.25	-
Zhangpuxiaosongdao	117°942′ E–24°177′ N	16	2	8	10	0.02	26.00	30.37	1.12	5.41	20.96	1142	0.35	1.39	-
Zhujiangdao	119°954′ E–26°776′ N	14	6	24	30	0.15	47.30	12.82	1.42	3.47	18.32	1589	0.81	8.83	0.66

**Table A2:** List of invasive alien plants on Fujian 53 islands.

Number	Family	Genus	Species
1	Acanthaceae	*Andrographis*	*Andrographis paniculata* (Burm. f.) Wall. ex Nees
2	Acanthaceae	*Justicia*	*Justicia gendarussa* Burm. f.
3	Aizoaceae	*Tetragonia*	*Tetragonia tetragonioides* (Pall.) Kuntze
4	Amaranthaceae	*Alternanthera*	*Alternanthera pungens* Kunth
5	Amaranthaceae	*Amaranthus*	*Amaranthus spinosus* L.
6	Amaranthaceae	*Celosia*	*Celosia cristata* L.
7	Amaranthaceae	*Dysphania*	*Dysphania ambrosioides* (L.) Mosyakin & Clemants
8	Amaranthaceae	*Alternanthera*	*Alternanthera philoxeroides* (Mart.) Griseb.
9	Amaranthaceae	*Amaranthus*	*Amaranthus tricolor* L.
10	Amaranthaceae	*Amaranthus*	*Amaranthus viridis* L.
11	Amaryllidaceae	*Zephyranthes*	*Zephyranthes candida* (Lindl.) Herb.
12	Amaryllidaceae	*Hippeastrum*	*Hippeastrum vittatum* (L’Hér.) Herb.
13	Apiaceae	*Cyclospermum*	*Cyclospermum leptophyllum* (Pers.) Sprague ex Britton & P. Wilson
14	Apocynaceae	*Asclepias*	*Asclepias curassavica* L.
15	Apocynaceae	*Catharanthus*	*Catharanthus roseus* (L.) G. Don
16	Asparagaceae	*Agave*	*Agave sisalana* Perr. ex Engelm.
17	Asparagaceae	*Agave*	*Agave americana* L.
18	Asteraceae	*Chromolaena*	*Chromolaena odorata* (L.) R. M. King & H. Rob.
19	Asteraceae	*Bidens*	*Bidens pilosa* L.
20	Asteraceae	*Sonchus*	*Sonchus asper* (L.) Hill
21	Asteraceae	*Ageratum*	*Ageratum conyzoides* L.
22	Asteraceae	*Praxelis*	*Praxelis clematidea* (Hieronymus ex Kuntze) R. M. King & H. Rob.
23	Asteraceae	*Coreopsis*	*Coreopsis lanceolata* L.
24	Asteraceae	*Rudbeckia*	*Rudbeckia laciniata* L.
25	Asteraceae	*Coreopsis*	*Coreopsis basalis* (A. Dietr.) S. F. Blake
26	Asteraceae	*Sonchus*	*Sonchus oleraceus* L.
27	Asteraceae	*Bidens*	*Bidens bipinnata* L.
28	Asteraceae	*Cosmos*	*Cosmos bipinnatus* Cav.
29	Asteraceae	*Erigeron*	*Erigeron sumatrensis* Retz.
30	Asteraceae	*Glebionis*	*Glebionis coronaria* (L.) Cass. ex Spach
31	Asteraceae	*Ambrosia*	*Ambrosia artemisiifolia* L.
32	Asteraceae	*Tagetes*	*Tagetes erecta* L.
33	Asteraceae	*Erigeron*	*Erigeron bonariensis* L.
34	Asteraceae	*Erigeron*	*Erigeron canadensis* L.
35	Asteraceae	*Erigeron*	*Erigeron annuus* (L.) Pers.
36	Asteraceae	*Pluchea*	*Pluchea sagittalis* (Lam.) Cabrera
37	Asteraceae	*Parthenium*	*Parthenium hysterophorus* L.
38	Asteraceae	*Tridax*	*Tridax procumbens* L.
39	Asteraceae	*Tithonia*	*Tithonia diversifolia* (Hemsl.) A. Gray
40	Asteraceae	*Symphyotrichum*	*Symphyotrichum subulatum* (Michx.) G. L. Nesom
41	Basellaceae	*Basella*	*Basella alba* L.
42	Bignoniaceae	*Macfadyena*	*Macfadyena unguis-cati* (L.) A.H.Gentry
43	Brassicaceae	*Lepidium*	*Lepidium virginicum* L.
44	Cactaceae	*Selenicereus*	*Selenicereus undatus* (Haw.) D.R.Hunt
45	Cactaceae	*Opuntia*	*Opuntia dillenii* (Ker Gawl.) Haw.
46	Caryophyllaceae	*Stellaria*	*Stellaria aquatica* (L.) Scop.
47	Caryophyllaceae	*Cerastium*	*Cerastium glomeratum* Thuill.
48	Casuarinaceae	*Casuarina*	*Casuarina equisetifolia* L.
49	Commelinaceae	*Tradescantia*	*Tradescantia zebrina* Bosse
50	Commelinaceae	*Tradescantia*	*Tradescantia pallida* (Rose) D. R. Hunt
51	Convolvulaceae	*Ipomoea*	*Ipomoea nil* (L.) Roth
52	Convolvulaceae	*Ipomoea*	*Ipomoea cairica* (L.) Sweet
53	Convolvulaceae	*Ipomoea*	*Ipomoea purpurea* (L.) Roth
54	Convolvulaceae	*Ipomoea*	*Ipomoea alba* L.
55	Crassulaceae	*Bryophyllum*	*Bryophyllum pinnatum* (L. f.) Oken
56	Cyperaceae	*Cyperus*	*Cyperus involucratus* Rottb.
57	Cyperaceae	*Cyperus*	*Cyperus rotundus* L.
58	Euphorbiaceae	*Euphorbia*	*Euphorbia maculata* L.
59	Euphorbiaceae	*Ricinus*	*Ricinus communis* L.
60	Euphorbiaceae	*Euphorbia*	*Euphorbia hirta* L.
61	Euphorbiaceae	*Pedilanthus*	*Pedilanthus tithymaloides* (L.) Poit.
62	Euphorbiaceae	*Jatropha*	*Jatropha curcas* L.
63	Euphorbiaceae	*Euphorbia*	*Euphorbia prostrata* Aiton
64	Euphorbiaceae	*Euphorbia*	*Euphorbia hypericifolia* L.
65	Malvaceae	*Malvastrum*	*Malvastrum coromandelianum* (L.) Garcke
66	Myrtaceae	*Eucalyptus*	*Eucalyptus robusta* Sm.
67	Euphorbiaceae	*Euphorbia*	*Euphorbia pulcherrima* Willd. ex Klotzsch
68	Fabaceae	*Robinia*	*Robinia pseudoacacia* L.
69	Fabaceae	*Acacia*	*Acacia auriculiformis* A. Cunn. ex Benth.
70	Fabaceae	*Mimosa*	*Mimosa pudica* L.
71	Fabaceae	*Acacia*	*Acacia mearnsii* De Wild.
72	Fabaceae	*Senna*	*Senna surattensis* (Burm. f.) H. S. Irwin & Barneby
73	Fabaceae	*Vachellia*	*Vachellia farnesiana* (L.) Wight & Arn.
74	Fabaceae	*Albizia*	*Albizia lebbeck* (L.) Benth.
75	Fabaceae	*Erythrina*	*Erythrina corallodendron L.*
76	Fabaceae	*Arachis*	*Arachis duranensis* Krapov. & W. C. Greg.
77	Fabaceae	*Cajanus*	*Cajanus cajan* (L.) Millsp.
78	Fabaceae	*Acacia*	*Acacia confusa* Merr.
79	Fabaceae	*Senna*	*Senna occidentalis* (L.) Link
80	Fabaceae	*Leucaena*	*Leucaena leucocephala* (Lam.) de Wit
81	Fabaceae	*Melilotus*	*Melilotus indicus* (L.) All.
82	Fabaceae	*Crotalaria*	*Crotalaria pallida* auct. non Aiton: T. L. Wu
83	Fabaceae	*Medicago*	*Medicago sativa* L.
84	Lamiaceae	*Mentha*	*Mentha spicata* L.
85	Lamiaceae	*Salvia*	*Salvia splendens* Ker Gawl.
86	Malvaceae	*Sida*	*Sida szechuensis* Matsuda
87	Malvaceae	*Abelmoschus*	*Abelmoschus esculentus* (L.) Moench
88	Poaceae	*Paspalum*	*Paspalum conjugatum* Bergius
89	Poaceae	*Cymbopogon*	*Cymbopogon citratus* (DC.) Stapf
90	Euphorbiaceae	*Euphorbia*	*Euphorbia cyathophora* Murray
91	Myrtaceae	*Psidium*	*Psidium guajava* L.
92	Nyctaginaceae	*Bougainvillea*	*Bougainvillea glabra* Choisy
93	Nyctaginaceae	*Bougainvillea*	*Bougainvillea spectabilis* Willd.
94	Nyctaginaceae	*Mirabilis*	*Mirabilis jalapa* L.
95	Onagraceae	*Oenothera*	*Oenothera drummondii* Hook.
96	Onagraceae	*Oenothera*	*Oenothera parviflora* L.
97	Onagraceae	*Oenothera*	*Oenothera biennis* L.
98	Orobanchaceae	*Orobanche*	*Orobanche brassicae* (Novopokr.) Novopokr.
99	Oxalidaceae	*Oxalis*	*Oxalis corymbosa* DC.
100	Papaveraceae	*Argemone*	*Argemone mexicana* L.
101	Papaveraceae	*Papaver*	*Papaver rhoeas* L.
102	Passifloraceae	*Passiflora*	*Passiflora edulis* Sims
103	Passifloraceae	*Passiflora*	*Passiflora caerulea* L.
104	Plantaginaceae	*Scoparia*	*Scoparia dulcis* L.
105	Plantaginaceae	*Plantago*	*Plantago lanceolata* L.
106	Poaceae	*Bromus*	*Bromus catharticus* Vahl
107	Poaceae	*Lolium*	*Lolium perenne* L.
108	Poaceae	*Melinis*	*Melinis repens* (Willd.) Zizka
109	Poaceae	*Chloris*	*Chloris virgata* Sw.
110	Poaceae	*Spartina*	*Spartina alterniflora* Loisel.
111	Poaceae	*Paspalum*	*Paspalum urvillei* Steud.
112	Poaceae	*Pennisetum*	*Pennisetum purpureum* Schumach.
113	Poaceae	*Cymbopogon*	*Cymbopogon nardus* (L.) Rendle
114	Poaceae	*Arrhenatherum*	*Arrhenatherum elatius* (L.) P. Beauv. ex J. Presl & C. Presl
115	Poaceae	*Avena*	*Avena fatua* L.
116	Polygonaceae	*Antigonon*	*Antigonon leptopus* Hook. & Arn.
117	Polygonaceae	*Muehlenbeckia*	*Muehlenbeckia platyclada* (F. Muell. ex Hook.) Meisn.
118	Pontederiaceae	*Eichhornia*	*Eichhornia crassipes* (Mart.) Solms
119	Portulacaceae	*Portulaca*	*Portulaca grandiflora* Hook.
120	Rubiaceae	*Spermacoce*	*Spermacoce alata* Aubl.
121	Solanaceae	*Physalis*	*Physalis peruviana* L.
122	Solanaceae	*Atropa*	*Atropa belladonna* L.
123	Solanaceae	*Solanum*	*Solanum erianthum* D. Don
124	Solanaceae	*Physalis*	*Physalis angulata* L.
125	Solanaceae	*Datura*	*Datura stramonium* L.
126	Solanaceae	*Solanum*	*Solanum capsicoides* All.
127	Solanaceae	*Cestrum*	*Cestrum nocturnum* L.
128	Urticaceae	*Pilea*	*Pilea microphylla* (L.) Liebm.
129	Verbenaceae	*Duranta*	*Duranta erecta* L.
130	Verbenaceae	*Lantana*	*Lantana camara* L.
